# PRDM1: a useful indicator of differentiation and prognosis in esophageal squamous cell carcinoma

**DOI:** 10.1186/s13000-026-01773-z

**Published:** 2026-02-28

**Authors:** Anqi Huang, Xingran Jiang, Yanan Qi, Jiaqi Chen, Xinmeng Guo, Jun Lu, Mulan Jin

**Affiliations:** Department of Pathology, Beijing Chao- yang Hospital, Capital Medical University, Beijing, 100020 China

**Keywords:** Esophageal squamous cell carcinoma, Histological grading, PRDM1, Tumor differentiation, Prognosis

## Abstract

**Background:**

Esophageal squamous cell carcinoma (ESCC) is a common malignant tumor of the gastrointestinal tract with a high mortality rate. Although positive regulatory domain zinc finger protein 1 (PRDM1) has long been thought to play a key role especially in the differentiation of B cells, its role in ESCC has never been studied. This study aimed to examine the association between PRDM1 and ESCC clinical and pathological characteristics and prognosis.

**Methods:**

Using immunohistochemical and reverse transcription quantitative polymerase chain reaction, we detected the expression level of PRDM1 in consecutive ESCC surgical resections from 163 patients. To interpret PRDM1 immunohistochemical positivity, we employed three methods: PRDM1 10 high-power field (HPF) positive cell count (PCC), PRDM1 1HPF PCC and PRDM1 positive hotspots (PPHs). Based on PPH assessment and histological morphology, we developed a novel histological grading scheme. To evaluate the relationship between PRDM1 differential expression and clinical–pathological parameters in ESCC, we used the chi-square test. To evaluate the relationship between PRDM1 expression and ESCC prognosis, we performed Kaplan–Meier survival analysis and Cox regression analysis.

**Results:**

Immunohistochemical staining revealed that PRDM1 was expressed in tumor epithelium, stroma, and adjacent squamous epithelium in ESCC. And the expression level of PRDM1 in tumor epithelium significantly correlated with tumor differentiation (*P* < 0.05) and was closely related to the patient prognosis (*P* < 0.05). Moreover, survival analysis results indicated that our novel histological grading seem to be better than the traditional histological grading criteria in predicting the prognosis of ESCC patients.

**Conclusions:**

PRDM1 holds promise as a novel indicator for ESCC, with potential application value in the histological grading, diagnosis, and prognostic assessment of this disease.

**Supplementary Information:**

The online version contains supplementary material available at 10.1186/s13000-026-01773-z.

## Introduction

Esophageal squamous cell carcinoma (ESCC) is a common, highly invasive malignant tumor that features prominently in global cancer statistics [1, 2]. Although existing evidence indicates that ESCC patients can derive survival benefits from radiotherapy, chemotherapy, and immunotherapy [3, 4], these adjuvant treatments have certain limitations [5–8]. Currently, the 5-year survival rate for ESCC patients remains below 20% [2, 9] The level of ESCC differentiation is usually assessed according to the 5th edition of the World Health Organization (WHO) Classification of Tumors of the Digestive System [10, 11]. However, differentiation according to WHO classification relies entirely on histological morphology, with most cases being classified as moderately differentiated. Furthermore, the clinical significance of histological grading of ESCC remains controversial [12–14]. Currently, there are no objective markers that can assist in the diagnosis of ESCC histological grading.

Positive regulatory domain zinc finger protein 1 (PRDM1) has been widely recognized as the primary regulatory protein for B-cell differentiation into plasma cells [15, 16, 17] Moreover, PRDM1 is also expressed in other lymphocytes [18–20] and plays a crucial role in embryonic development and tumorigenesis [21, 22] Nevertheless, few reports have focused on the role of PRDM1 in solid tumors [23–30]. Thus, the role of PRDM1 in ESCC has never been studied. To clarify its role in ESCC, this study aimed to investigate the expression of PRDM1 in normal esophageal epithelium and ESCC and examine its correlation with clinical pathological characteristics and prognosis.

## Materials and methods

### Patients and tissue samples

A total of 163 cases of surgical resection for ESCC diagnosed at Beijing Chaoyang Hospital, Capital Medical University, between January 2014 and January 2025 were collected. Tissue samples were collected from patients admitted for the first time who had not received prior radiotherapy, chemotherapy, or immunotherapy before or after surgery, and had undergone complete resection (pR0). Patients who did not meet these criteria, as well as those with stage I disease, were excluded. This study received approval from the Institutional Ethics Committee at Beijing Chaoyang Hospital, Capital Medical University. All cases were followed up on until May 2025. The follow-up data included age at initial diagnosis, status of surgical resection, and recurrence, metastasis, and/or death.

### Experimental methods

All pathological specimens submitted for examination were routinely fixed in 10% neutral formalin. Following fixation, the tissues underwent sampling, dehydration, paraffin embedding, and sectioning at a thickness of 3 to 4 μm with hematoxylin and eosin (HE) staining. Immunohistochemical (IHC) detection for all cases was performed using the EnVision method with DAB coloring. Anti-human PRDM1 rabbit monoclonal antibody (ab198287; 1:500 dilution; Abcam, Waltham, MA, USA) was used as the primary antibody. All procedures were strictly followed according to the reagent manual. Tonsil tissue served as the positive control, while phosphate-buffered saline was employed as the blank control in place of the primary antibody. Normal rabbit serum was used as the experimental control instead of the primary antibody to verify specific binding and eliminate non-specific interference.

### Histological evaluation

Tumors were classified as well differentiated (G1), moderately differentiated (G2), or poorly differentiated (G3) according to the histological criteria in the 5th edition of the WHO Classification of Tumors of the Digestive System [10, 11, 31]. Well-differentiated (G1) squamous cell carcinoma contains enlarged cells with abundant eosinophilic cytoplasm, keratin pearl production, minimal cytological atypia, a low mitotic count, an invasive margin, and cells that remain well-ordered. Moderately differentiated (G2) squamous cell carcinoma contains evident cytological atypia, fewer ordered cells, easily identified mitotic figures, frequent surface parakeratosis, and infrequent keratin pearl formation. Poorly differentiated (G3) squamous cell carcinoma consists predominantly of basal-like cells forming that may show central necrosis. The tumor nests consist of sheets or pavement-like arrangements of tumor cells with occasional parakeratotic or keratinizing cells.

According to the International Tumor Budding Consensus Conference criteria [32], each case was classified into one of the following three categories based on the number of tumor budding foci per high-power field (HPF): low-grade tumor budding: 0 to 4 tumor buds per HPF; intermediate-grade tumor budding: 5 to 9 tumor buds per HPF; and high-grade tumor budding: ≥10 tumor buds/HPF.

### Immunohistochemical staining interpretation

**Fig. 1:**
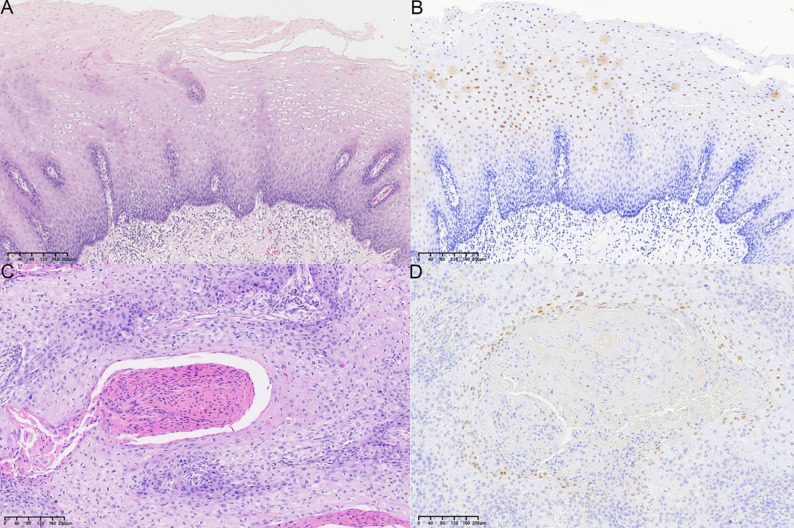
**A**, **B** Expression of normal squamous esophageal epithelium (**A**) and PRDM1: PRDM1 is expressed in the middle and superficial layers and not in the basal cell layer (**B**); **C**, **D** Expression pattern of PRDM1 in ESCC: PRDM1 is expressed in tumor cells around the keratinized pearl, while tumor cells at the periphery are either not expressed or weakly expressed. **A**, **C** HE stained sections; **B**, **D** Immunohistochemical stained sections

**Fig. 2:**
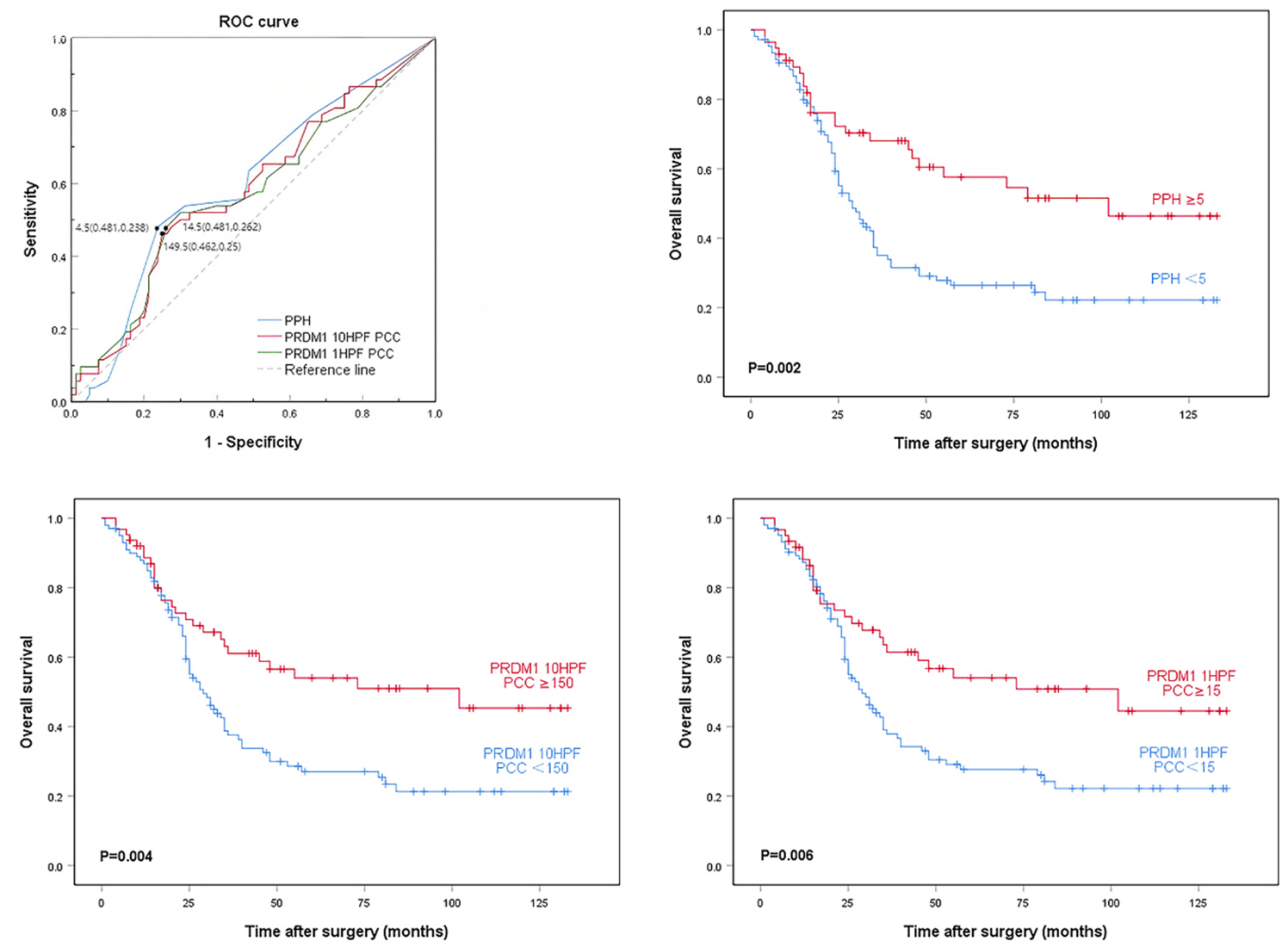
ROC curves for the 3 interpretation methods of PRDM1 expression levels and their relationship with overall survival. **A** ROC curves and cut-off values for the 3 interpretation methods of PRDM1; **B**–**D** Survival curve between the 3 interpretation methods and overall survival: PPH (**B**), PRDM1 1HPF PCC (**C**), and PRDM1 10HPF PCC (**D**)

Positive PRDM1 IHC staining is located in the nucleus. Three methods were used to interpret the degree of PRDM1 positivity: (1) PRDM1 10HPF positive cell count (PCC): Random consecutive fields of view were selected in the area of invasive cancer, and the number of positive tumor cells under 10HPF was counted and averaged. (2) PRDM1 1HPF PCC: Random consecutive fields of view were selected in the area of invasive cancer, and the number of positive tumor cells under 1HPF was counted and averaged. (3) PRDM1 positive hotspots (PPHs): Areas with 10 or more positive tumor cells continuously aggregating were defined as hotspots based on the average number of PRDM1 1HPF PCCs of all cases (Fig. 1). The PPH assessment workflow involves randomly scanning different selected fields of view at low magnification, evaluating and counting hotspot areas at medium-to-high magnification, counting the number of hotspot areas in 10× fields of view, and taking the average. All three methods were used to obtain the optimal critical value based on the receiver operating characteristic (ROC) curve (Fig. 2). Overall survival (OS) was selected as the primary endpoint for ROC-based cutoff optimization due to its status as the gold standard endpoint in oncologic prognosis. The corresponding optimal cutoff values in this experiment were 145 (10HPF PCC), 14.5 (1HPF PCC), and 5 (PPH). The AUCs of the three interpretation methods in this study were 0.700 (PPH), 0.680 (10HPF PCC), and 0.673 (1HPF PCC), suggesting that PRDM1 can be used as a predictor to classify ESCC. The cases were classified into PRDM1 high-expression and low-expression groups based on these cutoff values.

All available HE-stained and immunohistochemically-stained sections were reviewed and evaluated independently by two senior pathologists in a double-blind setting. In the event of discrepancies in the results, consultation was sought from a third authoritative pathologist to interpret the results and reach a conclusion.

### Development of a novel histological grading algorithm

**Fig. 3:**
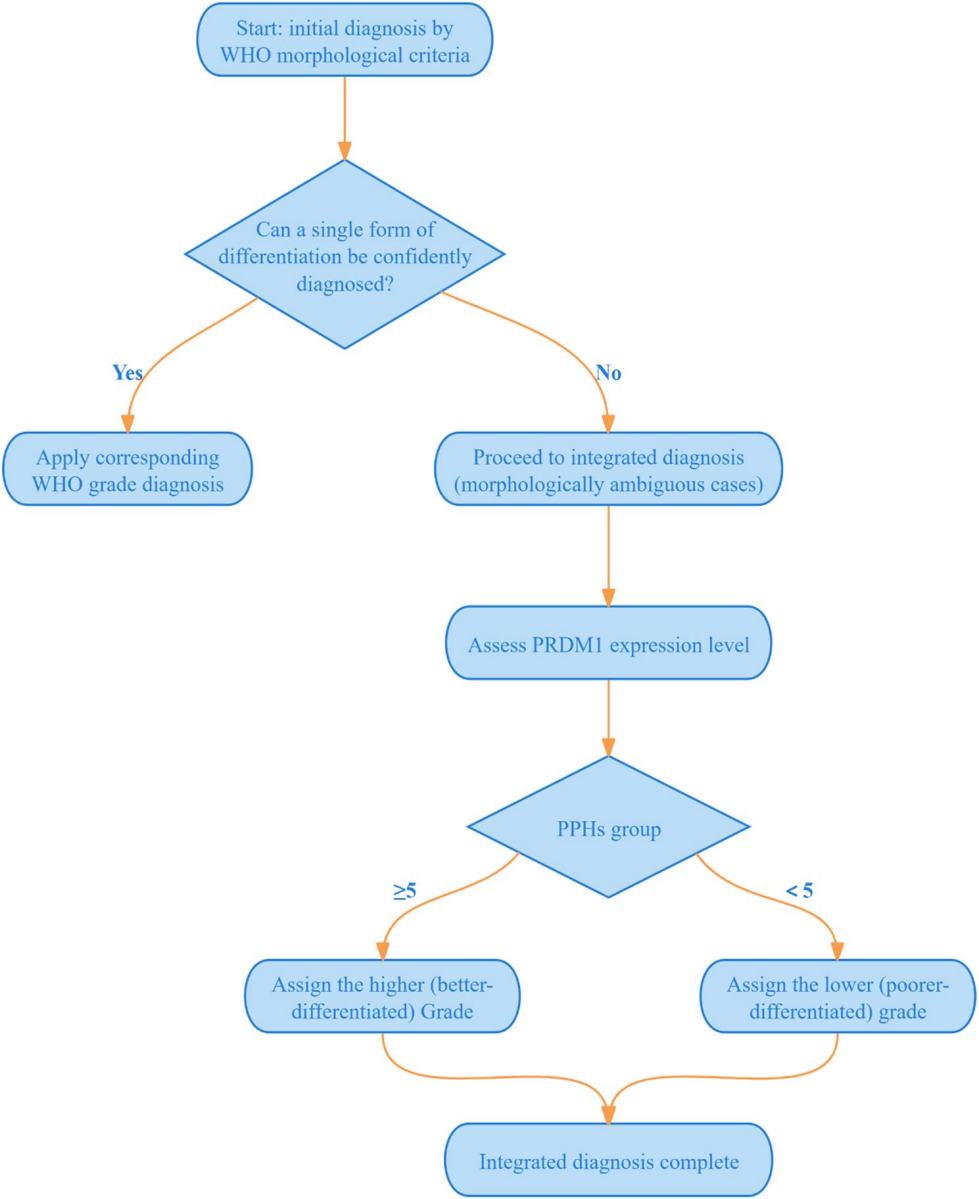
Flowchart of the PRDM1-integrated novel histological grading algorithm for ESCC differentiation

Based on our findings regarding PRDM1 expression level and the WHO histological morphology criteria, we developed a novel histological grading scheme to reassess the degree of tumor differentiation that we propose here. In brief, the algorithm proceeds as follows: first, conventional WHO criteria are applied; if a single differentiation pattern is clearly identifiable, the corresponding grade is assigned directly. When morphological features are ambiguous or overlap, PRDM1 immunohistochemistry is performed; high expression indicates a better differentiation grade, while low or negative expression suggests poorer differentiation. For example, a case judged as well-to-moderately differentiated by WHO criteria but whose exact subtype cannot be determined is ultimately classified as well differentiated if PRDM1 is highly expressed, or as moderately differentiated if PRDM1 is lowly expressed. To facilitate reproducibility, this decision algorithm was condensed into a stepwise flowchart (Fig. 3).

### Reverse transcription quantitative polymerase chain reaction (RT-qPCR) sample selection and processing

ESCC cases enrolled between January 2024 and January 2025 were screened for observation of HE sections. Cases with a tumor area proportion > 80% were selected, and tumor tissue and corresponding para-tumor esophageal tissue were selected from these cases, for a total of 18 paired samples. Laser capture microdissection (LCM) was used to separate tumor cells and stroma from the collected samples. Each FFPE tissue sample was sectioned into 8 to 12 slices of 4 μm thickness using a LEICA RM2135 microtome (Leica Microsystems, Germany), and the sections were placed in a LEICA HI1220 slide spreader (Leica Microsystems, Germany). These sections were then transferred to Leica membrane slides (Leica Microsystems, Germany). HE staining was performed using the fully automated LEICA AUTO STAINER XL staining instrument (Leica Microsystems, Germany), without covering the slides after staining. The stained slides were placed on the Leica LM7000 laser microdissection instrument (Leica Company, Germany) to cut the tumor cells and collect the tumor cell tissue fragments in centrifuge tubes.

### RT-qPCR experiment steps

RNA was extracted from collected tumor cell tissue fragments using the PureLink™ FFPE RNA Isolation Kit (K156002; Thermo Fisher Scientific, Waltham, MA, USA) according to standard procedures. RNA concentration and purity were detected using a NanoDrop (Yomim, Hangzhou, China, Unano-1000), and subsequently reverse-transcribed to cDNA using the Hibiscus for Genes reverse-transcription kit (11752050; ABI-invirtrogen, Waltham, MA, USA). DNA was amplified using the SYBR Green qPCR Master Mix buffer system (Servicebio, Wuhan, China, G3320) via a fluorescence quantitative PCR instrument (Long Gene, Hangzhou, China, Q200B). Glyceraldehyde-3-phosphate dehydrogenase was used as an indicator of RNA quality and the primer sequences were forward primer 5’-TCG GAG TCA ACG GAT TTG GTC GTA-3’ and reverse primer 5’-TGG CAT GGA CTG TGG TCA TGA GTC-3’ [33]. The PRDM1 primer sequences used in this study were forward primer 5’-GAT GCG GAT ATG ACT CTG TGG − 3’ and reverse primer 3’-CTC GGT TGC TTT AGA CTG CTC − 5‘ [34]. Quantitative gene expression levels were determined using the 2ΔΔCT method.

### Statistical analysis

Patients whose cause of death was unknown were reviewed at the date of the last assessment of death and the date of last contact. OS was defined as the time from surgical resection to death and included patients who were still alive at the last follow-up. All statistical tests were performed using IBM SPSS Statistics version 26. The χ2 test and Fisher’s exact test were used to assess differences between continuous and categorical variables. The sensitivity and specificity of indicators were analyzed using ROC curves. Kendall’s tau-b rank correlation coefficient was used to analyze correlations between continuous and categorical variables, with collinearity diagnostics performed for correlated variables. Cohen’s kappa statistic was calculated to assess agreement beyond chance, with 95% confidence intervals (CIs) computed. Survival curves were plotted using Kaplan-Meier analysis, univariate survival analyses were performed using log-rank tests, and analyses of prognostic influences were conducted using Cox proportional risk regression models for multifactorial analyses. For all analyses, a two-sided *P* < 0.05 was considered statistically significant.

## Results

### Patient clinicopathologic characteristics

The study included 163 patients with a median initial age of 63 years (range 41–85 years), the majority of whom were male (85.9%). Tumor lesions located in the low thoracic esophagus accounted for 66.2% of cases. The maximum diameter of the tumors ranged from 1.2 to 9.5 cm. There were no stage I patients. According to the Japanese Classification of Esophageal Cancer, 12th Edition, clinical TNM (cTNM) staging criteria, stage II predominated, accounting for 50.3% of cases, followed by stage III (81 cases, 49.7%); there were no patients with stage IV. According to the pathological TNM (pTNM) staging criteria, stage II predominated, accounting for 59.5% (97) of cases, followed by stage III (53 cases, 32.5%), and stage IV (13 cases, 9.8%). Regarding N stage, 62.0% of patients had lymph node metastases (N+). Nearly half (67.5%) exhibited moderate differentiation, while 9.8% and 22.7% exhibited well and poor differentiation, respectively. Tumor budding was detected in 91.4% of cases (149 cases), of which low grade was the most common (81 cases, 49.7%), followed by intermediate grade (65 cases, 39.8%) and high grade (17 cases, 10.4%). Table 1 summarizes the patient characteristics and their relationship with prognosis.

**Table 1:** Univariate analysis of 5-year survival rate of patients with ESCC

	*N* (%)	5-Year survival rate (%)	*P*
Gender			0.309
M	140 (85.9)	37.5	
F	23 (14.1)	36.6	
Age			0.204
< 65	92 (56.4)	41.8	
≥ 65	71 (43.6)	31.4	
Maximum tumor diameter			0.007**
< 4	82 (50.3)	21.5	
≥ 4	81 (49.7)	49.9	
Site			0.668
ME	55 (33.7)	32.0	
LE	108 (66.3)	39.4	
cTNM stage			0.010*
II	82 (50.3)	38.0	
III	81 (49.7)	13.2	
pTNM stage			0.007**
II	97 (59.3)	36.4	
III + IV	66 (40.7)	12.5	
WHO differentiation			0.091
Well (G1)/Moderate (G2)	126 (77.3)	41.1	
Poor (G3)	37 (22.7)	23.6	
WHO differentiation			0.155
Well (G1)	16 (9.8)	57.9	
Moderate (G2)	110 (67.5)	38.9	
Poor (G3)	37 (22.7)	23.6	
Novel histological grading			0.003**
Well (G1)	24 (14.7)	75.3	
Moderate (G2)	107 (65.6)	33.4	
Poor (G3)	32 (19.6)	22.1	
LVI			0.003**
No	56 (34.4)	54.7	
Yes	107 (65.6)	28.8	
Nerve invasion			< 0.001***
No	93 (57.1)	49.2	
Yes	70 (42.9)	22.2	
Tumor budding			0.009**
Low	81 (49.7)	50.0	
Intermediate + high	82 (51.3)	26.8	
pT stage			0.529
2	38 (23.3)	43.1	
3	125 (76.7)	35.9	
pN stage			< 0.001***
0	62 (38.0)	58.1	
1–3	101 (62.0)	25,2	
PRDM1 10HPF PCC group			0.004**
< 150	100 (61.3)	27.1	
≥ 150	63 (38.7)	54.0	
PRDM1 1HPF PCC group			0.006**
< 15	103 (63.2)	27.7	
≥ 15	60 (36.8)	54.0	
PPHs group			0.002**
< 5	106 (65.0)	26.4	
≥ 5	57 (35.0)	57.6	

*Abbreviations*: *F* Female, *M *Male, *HPF* High power field, *ME* Middle esophagus, *LE* Lower esophagus, *LVI* Lymphatic invasion or venous invasion, *PCC* Positive cell count, *PPH* PRDM1 positive hotspot

*Significant difference. *p < 0.05, **p < 0.01, ***P < 0,001

### Expression pattern of PRDM1 in esophageal peritumoral squamous epithelium and ESCC

The peritumoral squamous epithelium had measurable PRDM1 expression unevenly distributed among the stratified layers, with the highest expression in the superficial layers, which were also the most differentiated layers (Fig. 1). In ESCC, IHC staining showed intense PRDM1 immunoreactivity in the tumor cells adjacent to the keratin pearl, while the peripheral tumor cells showed no detectable levels of PRDM1 expression (Fig. 1). PRDM1 was likewise expressed in lymphocytes in the tumor mesenchyme.

### RT-qPCR validation

**Fig. 4:**
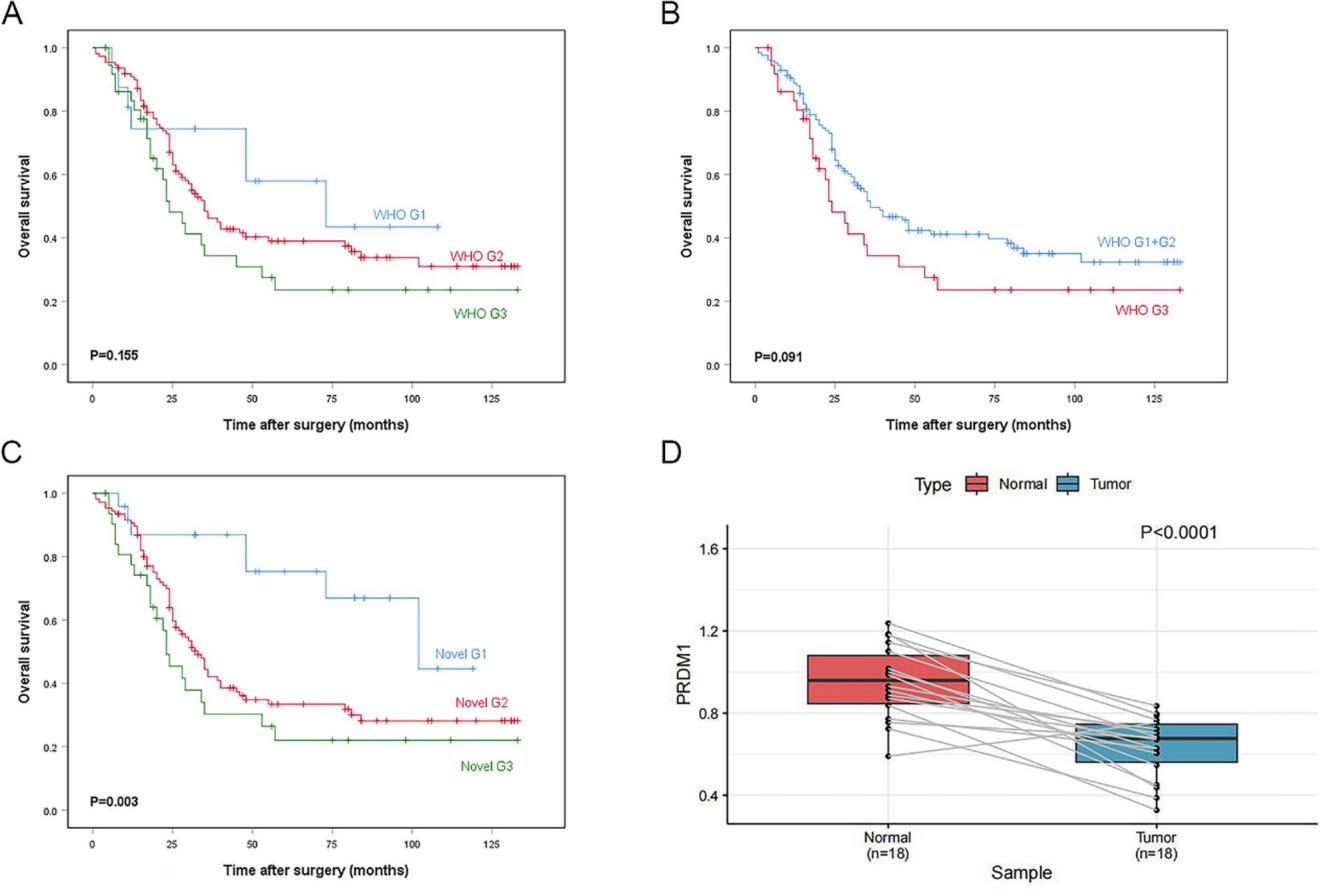
Survival curve between WHO histological grading and novel histological grading and overall survival in ESCC. **A**, **B** WHO histological grading; **C** Novel histological grading; **D** Expression level of PRDM1 in paired sample

RT-qPCR analysis of 18 paired ESCC tumor tissues and adjacent peritumoral epithelium revealed that PRDM1 mRNA expression was significantly downregulated in tumor cells compared with matched normal controls (*P* < 0.001, Fig. 4). These 18 paired samples are representative in terms of differentiation grade and TNM staging, reflecting the characteristics of the entire cohort. The validation cohort showed no significant differences from the entire cohort regarding age (*P* = 0.081), sex (*P* = 0.727), tumor size (*P* = 0.172), location (*P* = 0.972), differentiation grade (*P* = 0.325), cTNM stage (*P* = 0.980), pTNM stage (*P* = 0.556), and PRDM1 expression level (Supplementary Table S1).

### Clinicopathological characterization of PRDM1 and ESCC

**Fig. 5:**
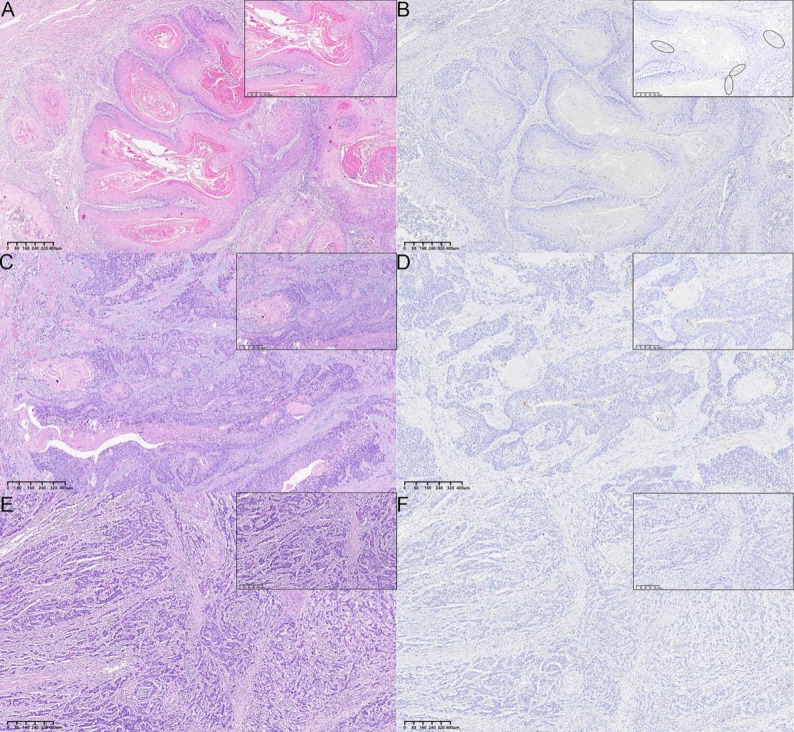
**A**, **B** Well differentiated ESCC: **A** Tumor contains numerous keratin pearls. Cytological atypia is minimal; **B** PRDM1 is highly expressed in tumor cells surrounding keratin pearls. The area circled in black represents PRDM1 hotspots; **C**, **D** Moderately differentiated ESCC: **C** Tumor shows marked cellular atypia, and a few keratin pearls; **D** PRDM1 is expressed in tumor cell surrounding a few keratin pearls; **E**, **F** Poorly differentiated ESCC: **E** Tumor shows significant atypia without keratinization pearls; **F** PRDM1 is minimal or absent. The inset shows cytological details under high magnification (40×). **A**, **C**, **E** HE-stained sections; **B**, **D**, **F** Immunohistochemical-stained section

The degree of PRDM1 positivity was assessed in each ESCC case using 3 interpretation methods, and PRDM1 expression was compared with the clinicopathological features of ESCC. These analyses revealed that PRDM1 expression in ESCC was inversely correlated with the histological grade (Fig. 5), with PRDM1 expression levels decreasing with a decrease in the degree of tumor differentiation (*P* = 0.002/0.003, Supplementary Table S2). PRDM1 expression was lower in stage III than in stage II cases in cTNM staging (*P* = 0.042/0.035/0.006) and lower in stage III + IV than in stage II cases in pTNM staging (*P* = 0.015/0.020/0.008). The PRDM1 expression level in the lymph node metastasis (N+) group was lower than that in the no lymph node metastasis (N0) group (*P* = 0.045/0.039/0.033). Further analysis of the correlation between PRDM1 expression and the degree of tumor differentiation showed that the PRDM1 expression level was significantly positively correlated with the degree of tumor differentiation (*P* = 0.010/0.012/0.016). These results indicate that low PRDM1 expression is closely associated with poor ESCC prognosis. However, no significant differences in PRDM1 expression levels between subgroups regarding other variables were observed (all *P* > 0.05, Supplementary Table S2). A comparison of the consistency of the three PRDM1 interpretation methods revealed a significant correlation among them (*P* < 0.001). Among the three assessment methods, the PPH group showed the most significant differences in TNM staging and lymph node metastasis (Supplementary Table S2).

### Association between PRDM1 and ESCC prognosis

To comprehensively observe the impact of PRDM1 expression on the prognosis of patients with ESCC, 163 cases were followed up, of which 138 had complete survival data, 25 were lost to follow-up, and 53.4% (*n* = 87) died from related or unrelated causes. The median survival time was 35 months, the median follow-up time was 81 months, and the 5-year OS rate was 37.2%. The results of univariate survival analysis showed that PRDM1 expression level, maximum tumor diameter, TNM stage, vascular invasion, nerve invasion, tumor budding, and lymph node metastasis were significantly associated with the 5-year survival rate in patients with ESCC (Table 1). The degree of tumor differentiation diagnosed according to WHO criteria was not associated with prognosis (*P* = 0.155, Fig. 4). As there were relatively few cases of well-differentiated ESCC in the cohort, they were combined with the cases of moderately differentiated ESCC and reanalyzed. The results of survival analysis after the combination showed that differentiation was still not significantly associated with prognosis (*P* = 0.091, Fig. 4).

Based on the results of univariate survival analysis, PPH was selected as the final interpretation for judging PRDM1 expression. OS statistics showed that the average survival time of patients with low PRDM1 expression was 50 months, with a 5-year survival rate of 26.4%, while the average survival time of patients in the high PRDM1 expression group was 81 months, with a 5-year survival rate of 57.6%. Kaplan-Meier survival analysis showed that patients with low PRDM1 expression had a worse OS than patients with high PRDM1 expression (Fig. 2). These results all indicate that reduced levels of PRDM1 expression in ESCC are detrimental to the prognosis of patients with ESCC.

### Application of the novel PRDM1-integrated grading scheme and its association with ESCC prognosis

When we reassessed the degree of differentiation in all cases using our novel histological grading scheme, we classified 24 samples as well differentiated, 107 as moderately differentiated, and 32 as poorly differentiated. Compared with the histological grading according to WHO criteria, the proportion of well-differentiated tumors increased (14.7%), and the proportion of moderately and poorly differentiated tumors decreased (65.6% and 19.6%, respectively). When we further evaluated the relationship between use of our novel histological grading scheme in diagnosis and ESCC prognosis by univariate survival analysis, we found that use of the novel scheme was significantly associated with the prognosis of patients with ESCC (*P* = 0.003, Table 1; Fig. 4).

### Multivariate analysis of OS

Based on the low P-value of the univariate survival analysis, maximal tumor diameter, novel histological grading, pTNM stage, lymphatic invasion or venous invasion, nerve invasion, tumor budding, and lymph node metastasis were selected to enter into the multivariate model. Multifactorial Cox regression analysis showed that maximal tumor diameter, vascular invasion nerve invasion and the novel histological grading scheme were independent prognostic factors affecting ESCC OS (Table 2).

**Table 2:** Multifactor Cox regression analysis model

	HR	95%CI	*P*
Maximum diameter of tumor	0.552	0.363–0.839	0.005**
pTNM stage	1.222	0.619–2.412	0.563
LVI	0.551	0.318–0.953	0.033*
Nerve invasion	1.735	1.089–2.764	0.020*
Tumor Budding	0.676	0.318–1.434	0.307
N stage	0.763	0.479–1.215	0.254
Novel histological grading	1.555	1.098–2.202	0.013*

*Abbreviations*: *HR* Hazard ratios, *CI* Confidence intervals, *LVI* Lymphatic invasion or venous invasion

*Significant difference. *p < 0.05, **p < 0.01

## Discussion

To the best of our knowledge, this study is the first investigation to explore the potential role of PRDM1 in ESCC. The results demonstrate that PRDM1 expression level is significantly correlated with the degree of ESCC tumor differentiation and ESCC prognosis. Therefore, we propose a novel histological grading scheme for ESCC that is more highly correlated with ESCC prognosis than ESCC histological grading based on WHO guidelines.

PRDM1 is an inhibitory protein with a zinc finger domain that was first discovered as a silencer to block β-interferon gene expression [35], which is also known as B lymphocyte induced maturation protein 1 (Blimp-1) in mice [16, 36]. A key molecule of B-lymphocyte differentiation, PRDM1 prolongs the plasma cell stage and promotes the secretion of immunoglobulin. Studies have shown that PRDM1 also acts on T cells [19], NK cells [20], and macrophages [18]. 

PRDM1 also plays an important role in tumorigenesis [37] and in the early development of some tissues [15]. However, most studies of PRDM1 and tumors have involved hematological tumors, and only a few reports have examined the role of PRDM1 in solid tumors [23, 25]. In our study, the first report of the role of PRDM1 in ESCC, we confirmed the expression of PRDM1 in ESCC by RT-qPCR and IHC staining. Our RT-qPCR results suggest that PRDM1 gene expression is downregulated in ESCC tumor epithelium compared with esophageal peritumoral squamous epithelium (Fig. 4). Although the RT-qPCR validation was conducted on a relatively small sample size (*n* = 18), these cases were representative of the overall cohort based on clinicopathological characteristics. However, the limited sample size remains a constraint, and further validation in larger independent cohorts is warranted.

In this study, we collected ESCC samples for IHC staining to observe the expression of PRDM1 protein in ESCC tumor epithelium. In previous studies, IHC staining of PRDM1 expression level was mainly performed to assess the area occupied by the number of positive cells [23, 38, 39]. However, as PRDM1 is expressed in both tumor cells and lymphocytes in the mesenchyme, this method is susceptible to interference by positive cells in the mesenchyme. Therefore, the three different interpretation methods we used all counted PRDM1-positive tumor cells, which can more accurately assess the expression of PRDM1 protein in tumor cells. Moreover, our RT-qPCR results confirmed the presence of PRDM1 gene amplification in tumor cells in ESCC.

In our research, we observed that the expression pattern of PRDM1 in the epithelium of ESCC tumors was primarily in the form of small foci around the keratinized pearls, and most of the ESCC tumor tissues took the form of nests of varying sizes. Based on the expression pattern, we used PPH assessment, for the first time, as a method to identify PRDM1. Using this method, we were able to identify the expression level of PRDM1 more easily and directly compared with PRDM1 10HPF PCC and 1HPF PCC. The kappa value of the PPH method was 0.848 (95% CI: 0.756–0.940, *P* < 0.001), indicating excellent agreement. Statistical analysis showed that there was no significant difference in P-values among the three PRDM1 interpretation methods with respect to tumor differentiation; however, the PPH method performed better in TNM staging, lymph node metastasis, and nerve invasion. Furthermore, the PPH method exhibits a higher AUC value. Therefore, we propose that the PPH interpretation method is superior in assessing the prognosis of patients with ESCC because it can more intuitively and rapidly interpret the PRDM1 protein expression level, and we therefore recommend its use as an interpretation method.

Some studies have shown that the PRDM1 gene is a key regulator of terminal differentiation in epidermal keratinocytes, particularly during the differentiation of granular layer cells into stratum corneum cells [22]. PRDM1 is important for the differentiation of a variety of tissues [15]. Previous studies have demonstrated that PRDM1 can be expressed in normal squamous epithelium [40]. However, no studies to date have investigated the association between PRDM1 and squamous cell carcinoma differentiation. Our study is the first to show that PRDM1 expression levels in ESCC were significantly and positively correlated with the degree of tumor differentiation (*P* = 0.010/0.012/0.016, Supplementary Table S2). As the extent of tumor differentiation increased, the level of PRDM1 protein expression increased, which we attributed to PRDM1 expression in the terminally differentiated cells around the keratinized pearls in ESCC. Observing that assessment of PRDM1 expression can more accurately identify well-differentiated areas in the tumor, we propose that assessment of PRDM1 expression provides a more objective determination of the degree of tumor differentiation.

Currently, the histological grading of ESCC is mainly assessed according to the 5th edition of the WHO Classification of Tumors of the Digestive System [10, 11]. However, the WHO-based histological grading of ESCC has been controversial, with some studies indicating that the aggressive biological behavior and prognosis of ESCC are closely related to the degree of differentiation of the tumor [12] and others showing fundamental weaknesses in determining the prognosis of patients, not only in ESCC but also in a wide variety of other tumors [9, 13, 41]. The results of survival analysis in this study similarly showed that tumor differentiation diagnosed according to histological morphology did not correlate with OS in patients with ESCC (*P* = 0.155, Table 1). When we combined cases with highly differentiated and moderately differentiated tumors and performed survival analysis, we again did not find a statistically significant correlation. However, we identified a trend suggesting that poor tumor differentiation may indicate a poor prognosis in patients with ESCC (*P* = 0.091). This may be a result of the WHO criteria being highly based on subjective judgment, and the difficulty in distinguishing between well-differentiated and moderately differentiated cases. Of the 163 cases in our study, only 16 cases (9.8%) were classified as well differentiated, with 110 cases (67.5%) diagnosed as moderately differentiated. Therefore, our findings suggest that traditional histological grading based on solely histological morphology diagnosis has poor accuracy for predicting the prognosis of ESCC.

Reassessing histological grading using PPH combined with histological morphology by using our novel histological grading scheme, we found that the number of well-differentiated cases increased to 24, and the number of moderately differentiated cases decreased to 107. Further survival analyses showed that use of the novel histological grading scheme was significantly correlated with OS (*P* = 0.003, Fig. 4). Multivariable Cox regression analysis revealed that use of the novel histological grading scheme was an independent factor influencing the prognosis of ESCC (*P* = 0.013, Table 2). These findings suggest that the detection of PRDM1 can help determine the histological grading more accurately and that use of the novel histological grading scheme can effectively predict the prognosis of ESCC. Thus, PRDM1 can be used as an indicator for determining the extent of malignancy in ESCC and has some reference value in guiding clinical diagnosis.

Our results showed that in addition to correlating with the degree of differentiation, PRDM1 protein expression level was also closely associated with TNM stage and lymph node metastasis, suggesting that ESCC cases with high PRDM1 expression have a lower TNM stage and are less prone to lymph node metastasis than cases with low or no PRDM1 expression. This also indicates that the level of PRDM1 expression can predict the degree of invasiveness and metastasis and, to a certain extent, the prognosis of ESCC. Detecting the PRDM1 expression level in ESCC tissues can help evaluate the invasiveness and other biological behaviors of ESCC from another side, assisting in the clinical selection of high-risk cases and guiding the comprehensive diagnosis and treatment of ESCC.

Although our study yielded several significant findings, it is subject to certain limitations. In addition to its retrospective nature, our study is limited by the fact that our analyses are currently restricted to resection specimens without neoadjuvant treatment. As our sample was limited in terms of diversity, our results must be validated in larger ESCC cohorts within a variety of different clinical and pathologic settings. Similarly, as an investigation into tumor prognostic markers, this study did not further explore functional evidence [42]. To overcome this limitation, we will conduct in vivo and in vitro experiments in subsequent work to continue investigating the function and molecular mechanisms of PRDM1 in ESCC.

In summary, our findings suggest that the expression of PRDM1 in the ESCC tumor epithelium is closely associated with tumor differentiation and patient prognosis. Furthermore, the novel histological grading scheme that we proposed has significant prognostic relevance for ESCC and is valuable in guiding tumor differentiation diagnosis. Therefore, we suggest that PRDM1 holds promise as a novel indicator for ESCC, with potential application value in the histological grading, diagnosis, and prognostic assessment of this disease.

## Supplementary Information

Additional file 1: Table S1: Comparison of Clinical Pathological Characteristics between the RT-qPCR Cohort and Overall Cohort. Table S2: Correlation Analysis of Clinical Pathological Characteristics and PRDM1 in Patients With ESCC.

## Data Availability

The data used to support the results of this study can be obtained from the corresponding author.

## Abbreviations

AUC: Area under curve
CI: Confidence interval
ESCC: Esophageal squamous cell carcinoma
FFPE: Formalin fixed paraffin embedded
HE: Hematoxylin and eosin
HPF: High-power field
IHC: Imunohistochemical
LCM: Laser capture microdissection
OS: Overall survival
PCC: Positive cell count
PRDM1: Positive regulatory domain zinc finger protein 1
PPH: PRDM1 positive hotspots
ROC: Receiver operating characteristic
RT-qPCR: Reverse transcription quantitative polymerase chain reaction
WHO: World Health Organization

## References

[1] He S, Xu J, Liu X, Zhen Y. Advances and challenges in the treatment of esophageal cancer. Acta Pharm Sin B. 2021;11:3379–92. 10.1016/j.apsb.2021.03.008. 34900524 10.1016/j.apsb.2021.03.008 PMC8642427

[2] Abnet CC, Arnold M, Wei W-Q. Epidemiology of esophageal squamous cell carcinoma. Gastroenterology. 2018;154:360–73. 10.1053/j.gastro.2017.08.023. 28823862 10.1053/j.gastro.2017.08.023 PMC5836473

[3] Guo X, Chen C, Zhao J, Wang C, Mei X, Shen J, et al. Neoadjuvant chemoradiotherapy vs chemoimmunotherapy for esophageal squamous cell carcinoma. JAMA Surg. 2025;160:565–74. 10.1001/jamasurg.2025.0220. 40105813 10.1001/jamasurg.2025.0220 PMC11923775

[4] Yang H, Liu H, Chen Y, Zhu C, Fang W, Yu Z, et al. Long-term efficacy of neoadjuvant chemoradiotherapy plus surgery for the treatment of locally advanced esophageal squamous cell carcinoma: the NEOCRTEC5010 randomized clinical trial. JAMA Surg. 2021;156:721–9. 10.1001/jamasurg.2021.2373. 34160577 10.1001/jamasurg.2021.2373 PMC8223138

[5] Zhang H, Shi Y, Ying J, Chen Y, Guo R, Zhao X, et al. A bibliometric and visualized research on global trends of immune checkpoint inhibitors related complications in melanoma, 2011–2021. Front Endocrinol. 2023;14:1164692. 10.3389/fendo.2023.1164692. 10.3389/fendo.2023.1164692 PMC10158729 37152956

[6] Zhang B, Lau LY, Chen Y, Xie R. Bibliometric analysis of immune-related acute kidney injury induced by cancer immunotherapy (2000–2025). Naunyn Schmiedebergs Arch Pharmacol. 2025. 10.1007/s00210-025-04582-1. 40924107 10.1007/s00210-025-04582-1

[7] Zhang B, Lau LY, Wu Z, Chen Y. Chemotherapy induced neurotoxicity in cancer survivors assessed through a dual database bibliometric analysis from 2005 to 2025. Discov Oncol. 2025;16:1961. 10.1007/s12672-025-03802-7. 41134460 10.1007/s12672-025-03802-7 PMC12552212

[8] Wu Z, Chen Y, Yu G, Ma Y. Research trends and hotspots in surgical treatment of recurrent nasopharyngeal carcinoma: a bibliometric analysis from 2000 to 2023. Asian J Surg. 2024;47:2939–41. 10.1016/j.asjsur.2024.02.106. 38431480 10.1016/j.asjsur.2024.02.106

[9] Wang J, Wu N, Zheng Q-F, Yan S, Lv C, Li S-L, et al. Evaluation of the 7th edition of the TNM classification in patients with resected esophageal squamous cell carcinoma. World J Gastroenterol. 2014;20:18397–403. 10.3748/wjg.v20.i48.18397. 25561808 10.3748/wjg.v20.i48.18397 PMC4277978

[10] Lam AK-Y. Updates on world health organization classification and staging of esophageal tumors: implications for future clinical practice. Hum Pathol. 2021;108:100–12. 10.1016/j.humpath.2020.10.015. 33157124 10.1016/j.humpath.2020.10.015

[11] Rice TW, Gress DM, Patil DT, Hofstetter WL, Kelsen DP, Blackstone EH. Cancer of the esophagus and esophagogastric junction-major changes in the American joint committee on cancer eighth edition cancer staging manual. CA Cancer J Clin. 2017;67:304–17. 10.3322/caac.21399. 28556024 10.3322/caac.21399

[12] Wang LS, Chow KC, Chi KH, Liu CC, Li WY, Chiu JH, et al. Prognosis of esophageal squamous cell carcinoma: analysis of clinicopathological and biological factors. Am J Gastroenterol. 1999;94:1933–40. 10.1111/j.1572-0241.1999.01233.x. 10406262 10.1111/j.1572-0241.1999.01233.x

[13] Edwards JM, Hillier VF, Lawson RA, Moussalli H, Hasleton PS. Squamous carcinoma of the oesophagus: histological criteria and their prognostic significance. Br J Cancer. 1989;59:429–33. 10.1038/bjc.1989.87. 2930710 10.1038/bjc.1989.87 PMC2247087

[14] Deng J, Zhang L, Wang Z, Li B, Xiang J, Ma L, et al. Pathological features of the differentiation landscape in esophageal squamous cell cancer and their correlations with prognosis. Front Oncol. 2024;14:1442212. 10.3389/fonc.2024.1442212. 39711958 10.3389/fonc.2024.1442212 PMC11659131

[15] John SA, Garrett-Sinha LA. Blimp1: a conserved transcriptional repressor critical for differentiation of many tissues. Exp Cell Res. 2009;315:1077–84. 10.1016/j.yexcr.2008.11.015. 19073176 10.1016/j.yexcr.2008.11.015

[16] Turner CA, Mack DH, Davis MM. Blimp-1, a novel zinc finger-containing protein that can drive the maturation of B lymphocytes into immunoglobulin-secreting cells. Cell. 1994;77:297–306. 10.1016/0092-8674(94)90321-2. 8168136 10.1016/0092-8674(94)90321-2

[17] Angelin-Duclos C, Cattoretti G, Lin KI, Calame K. Commitment of B lymphocytes to a plasma cell fate is associated with blimp-1 expression in vivo. J Immunol Baltim Md 1950. 2000;165:5462–71. 10.4049/jimmunol.165.10.5462. 10.4049/jimmunol.165.10.5462 11067898

[18] Doody GM, Stephenson S, Tooze RM. BLIMP-1 is a target of cellular stress and downstream of the unfolded protein response. Eur J Immunol. 2006;36:1572–82. 10.1002/eji.200535646. 16708403 10.1002/eji.200535646

[19] Xin A, Nutt SL, Belz GT, Kallies A. Blimp1: driving terminal differentiation to a T. Adv Exp Med Biol. 2011;780:85–100. 10.1007/978-1-4419-5632-3_8. 21842367 10.1007/978-1-4419-5632-3_8

[20] Smith MA, Maurin M, Cho HI, Becknell B, Freud AG, Yu J, et al. PRDM1/blimp-1 controls effector cytokine production in human NK cells. J Immunol Baltim Md 1950. 2010;A185:6058–67. 10.4049/jimmunol.1001682. 10.4049/jimmunol.1001682 PMC3864810 20944005

[21] Vincent SD, Dunn NR, Sciammas R, Shapiro-Shalef M, Davis MM, Calame K, et al. The zinc finger transcriptional repressor Blimp1/Prdm1 is dispensable for early axis formation but is required for specification of primordial germ cells in the mouse. Dev Camb Engl. 2005;132:1315–25. 10.1242/dev.01711. 10.1242/dev.01711 15750184

[22] Magnúsdóttir E, Kalachikov S, Mizukoshi K, Savitsky D, Ishida-Yamamoto A, Panteleyev AA, et al. Epidermal terminal differentiation depends on B lymphocyte-induced maturation protein-1. Proc Natl Acad Sci. 2007;104:14988–93. 10.1073/pnas.0707323104. 17846422 10.1073/pnas.0707323104 PMC1986600

[23] Wang L, Zhang W-P, Yao L, Zhang W, Zhu J, Zhang W-C, et al. PRDM1 expression via human parvovirus B19 infection plays a role in the pathogenesis of Hashimoto thyroiditis. Hum Pathol. 2015;46:1913–21. 10.1016/j.humpath.2015.08.009. 26475096 10.1016/j.humpath.2015.08.009

[24] Li Q, Zhang L, You W, Xu J, Dai J, Hua D, et al. PRDM1/BLIMP1 induces cancer immune evasion by modulating the USP22-SPI1-PD-L1 axis in hepatocellular carcinoma cells. Nat Commun. 2022;13:7677. 10.1038/s41467-022-35469-x. 36509766 10.1038/s41467-022-35469-x PMC9744896

[25] Ma J, Li Z, Xu J, Lai J, Zhao J, Ma L, et al. PRDM1 promotes the ferroptosis and immune escape of thyroid cancer by regulating USP15-mediated SELENBP1 deubiquitination. J Endocrinol Invest. 2024;47:2981–97. 10.1007/s40618-024-02385-4. 39014173 10.1007/s40618-024-02385-4

[26] Nong L, Zheng Y, Li X, Li D, Liang L, Wang W, et al. The genetic deletion and protein expression of PRDM1 and its clinical implications in diffuse large B cell lymphoma: a retrospective cohort study in China. Pathol Res Pract. 2022;233:153860. 10.1016/j.prp.2022.153860. 35429891 10.1016/j.prp.2022.153860

[27] Tang J, Li Y, Wu J, Shen H, Yin H, Liang J, et al. Bortezomib depended on PRDM1 and TP53 to exert therapeutic effect in activated B-cell-like diffuse large B-cell lymphoma. Genes Dis. 2024;11:550–3. 10.1016/j.gendis.2023.04.012. 37692509 10.1016/j.gendis.2023.04.012 PMC10491910

[28] Qing K, Jin Z, Xu Z, Wang W, Li X, Zhang Y, et al. Dysregulated MDR1 by PRDM1/Blimp1 is involved in the doxorubicin resistance of non-germinal center B-cell-like diffuse large B-cell lymphoma. Chemotherapy. 2022;67:12–23. 10.1159/000520070. 34844236 10.1159/000520070

[29] Yu C, Li J, Kuang W, Ni S, Cao Y, Duan Y. PRDM1 promotes nucleus pulposus cell pyroptosis leading to intervertebral disc degeneration via activating CASP1 transcription. Cell Biol Toxicol. 2024;40:89. 10.1007/s10565-024-09932-y. 39432156 10.1007/s10565-024-09932-y PMC11493826

[30] Guo H, Wang M, Wang B, Guo L, Cheng Y, Wang Z, et al. PRDM1 drives human primary T cell hyporesponsiveness by altering the T cell transcriptome and epigenome. Front Immunol. 2022;13:879501. 10.3389/fimmu.2022.879501. 35572579 10.3389/fimmu.2022.879501 PMC9097451

[31] Rice TW, Ishwaran H, Ferguson MK, Blackstone EH, Goldstraw P. Cancer of the esophagus and esophagogastric junction: an eighth edition staging primer. J Thorac Oncol Off Publ Int Assoc Study Lung Cancer. 2017;12:36–42. 10.1016/j.jtho.2016.10.016. 10.1016/j.jtho.2016.10.016 PMC5591443 27810391

[32] Lugli A, Kirsch R, Ajioka Y, Bosman F, Cathomas G, Dawson H et al. Recommendations for reporting tumor budding in colorectal cancer based on the international tumor budding consensus conference (ITBCC) 2016. Mod Pathol Off J U S Can Acad Pathol Inc. 2017;30:1299–311. 10.1038/modpathol.2017.46. 10.1038/modpathol.2017.46 28548122

[33] Zhao X, Carnevale KA, Cathcart MK. Human monocytes use Rac1, not Rac2, in the NADPH oxidase complex. J Biol Chem. 2003;278:40788–92. 10.1074/jbc.M302208200. 12912997 10.1074/jbc.M302208200

[34] Bryant VL, Ma CS, Avery DT, Li Y, Good KL, Corcoran LM, et al. Cytokine-mediated regulation of human B cell differentiation into ig-secreting cells: predominant role of IL-21 produced by CXCR5 + T follicular helper cells. J Immunol Baltim Md 1950. 2007;179:8180–90. 10.4049/jimmunol.179.12.8180. 10.4049/jimmunol.179.12.8180 18056361

[35] Keller AD, Maniatis T. Identification and characterization of a novel repressor of beta-interferon gene expression. Genes Dev. 1991;5:868–79. 10.1101/gad.5.5.868. 1851123 10.1101/gad.5.5.868

[36] Huang S. Blimp-1 is the murine homolog of the human transcriptional repressor PRDI-BF1. Cell. 1994;78:9. 10.1016/0092-8674(94)90565-7. 8033216 10.1016/0092-8674(94)90565-7

[37] Tang TF, Chan YT, Cheong HC, Cheok YY, Anuar NA, Looi CY, et al. Regulatory network of BLIMP1, IRF4, and XBP1 triad in plasmacytic differentiation and multiple myeloma pathogenesis. Cell Immunol. 2022;380:104594. 10.1016/j.cellimm.2022.104594. 36081178 10.1016/j.cellimm.2022.104594

[38] Klein M, Vignaud JM, Hennequin V, Toussaint B, Bresler L, Plénat F, et al. Increased expression of the vascular endothelial growth factor is a pejorative prognosis marker in papillary thyroid carcinoma. J Clin Endocrinol Metab. 2001;86:656–8. 10.1210/jcem.86.2.7226. 11158026 10.1210/jcem.86.2.7226

[39] Guo Y-G, Zhang L-L, Hu P, Li Z-Z, Zhang R-B, Lv X, et al. Correlation analysis of bone marrow microvessel density and MiRNA expression on drug resistance in patients with chronic myelogenous leukemia after tyrosine kinase inhibitor treatment. Hematol Amst Neth. 2024;29:2304488. 10.1080/16078454.2024.2304488. 10.1080/16078454.2024.2304488 38299685

[40] Garcia J-F, Roncador G, García J-F, Sánz A-I, Maestre L, Lucas E, et al. PRDM1/BLIMP-1 expression in multiple B and T-cell lymphoma. Haematologica. 2006;91:467–74. 16585013

[41] Sarbia M, Bittinger F, Porschen R, Dutkowski P, Willers R, Gabbert HE. Prognostic value of histopathologic parameters of esophageal squamous cell carcinoma. Cancer. 1995;76:922–7. 10.1002/1097-0142(19950915)76:6<922. ::aid-cncr2820760603%253E3.0.co;2-q. 10.1002/1097-0142(19950915)76:6<922::aid-cncr2820760603>3.0.co;2-q 8625216

[42] Liu Y, Chen Y, Wang F, Lin J, Tan X, Chen C, et al. Caveolin-1 promotes glioma progression and maintains its mitochondrial Inhibition resistance. Discov Oncol. 2023;14:161. 10.1007/s12672-023-00765-5. 37642765 10.1007/s12672-023-00765-5 PMC10465474

