# Supplementary material for: PRDM1: a useful indicator of differentiation and prognosis in esophageal squamous cell carcinoma
Source: Diagn Pathol. 2026 Feb 28;21:33. doi: 10.1186/s13000-026-01773-z (PMC13059383; doi:10.1186/s13000-026-01773-z)
Supplement: Supplementary file 1 — Additional file 1: Table S1: Comparison of Clinical Pathological Characteristics between the RT-qPCR Cohort and Overall Cohort. Table S2: Correlation Analysis of Clinical Pathological Characteristics and PRDM1 in Patients With ESCC. [file 13000_2026_1773_MOESM1_ESM.pdf]

Supplementary Table S1

Table S1. Comparison of Clinical Pathological Characteristics between the RT-qPCR Cohort and Overall Cohort

	**Overall cohort**	**RT-qPCR cohort**	***P***
N (%)	163 (100)	18 (100)	
Gender			0.727
M	140 (85.9)	15 (83.3)	
F	23 (14.1)	3 (16.7)	
Age			0.081
<65	92 (56.4)	6 (33.3)	
≥65	71 (43.6)	12 (66.7)	
Tumor size			0.172
<4	82 (50.3)	6 (33.3)	
≥4	81 (49.7)	12 (66.7)	
Site			0.972
ME	55 (33.7)	6 (33.3)	
LE	108 (66.3)	12 (66.7)	
cTNM stage			0.980
II	82 (50.3)	9 (50)	
III	81 (49.7)	9 (50)	
pTNM stage			0.556
II	97 (59.3)	12 (66.7)	
III + IV	66 (40.7)	6 (33.3)	
WHO Differentiation			0.325
Well (G1)	16 (9.8)	3 (16.7)	
Moderate (G2)	110 (67.5)	9 (50)	
Poor (G3)	37 (22.7)	6 (33.3)	

PRDM1 10HPF PCC group			0.659
<150	100 (61.3)	12 (66.7)	
≥150	63 (38.7)	6 (33.3)	
PRDM1 1HPF PCC group			0.771
<15	103 (63.2)	12 (66.7)	
≥15	60 (36.8)	6 (33.3)	
PPHs group			0.890
<5	106 (65.0)	12 (66.7)	
≥5	57 (35.0)	6 (33.3)	

Abbreviations: F, female; M, male; HPF, high power field; ME, middle esophagus; LE, lower esophagus; PCC, positive cell count; PPH, PRDM1 positive hotspot.

Supplementary Table S2

Table S2. Correlation Analysis of Clinical Pathological Characteristics and PRDM1 in Patients With ESCC

	**N**	**PRDM1 10HPF PCC group**		***P***	**PRDM1 1HPF PCC group**		***P***	**PPHs group**		***P***
		<150 (%)	≥150 (%)		<15 (%)	≥15 (%)		<5 (%)	≥5 (%)	
N		100 (61.3)	63 (38.7)		103 (63.2)	60 (36.8)		106 (65.0)	57 (35.0)	
Gender				0.959			0.819			0.652
M	140	86 (61.4)	54 (38.6)		89 (63.6)	51 (36.4)		92 (65.7)	48 (34.3)	
F	23	14 (60.9)	9 (39.1)		14 (60.9)	9 (39.1)		14 (60.9)	9 (39.1)	
Age				0.886			0.745			0.784
<65	92	56 (60.9)	36 (39.1)		57 (62.0)	35 (38.0)		59 (64.1)	33 (35.9)	
≥65	71	44 (62.0)	27 (38.0)		46 (64.8)	25 (35.2)		47 (66.2)	24 (33.8)	
Tumor size				0.424			0.196			0.380
<4	82	53 (64.6)	29 (35.4)		56 (68.3)	26 (31.7)		56 (68.3)	26 (31.7)	
≥4	81	47 (58.0)	34 (42.0)		47 (58.0)	34 (42.0)		50 (61.7)	31 (38.3)	
Site				0.175			0.087			0.668

ME	55	38 (69.1)	17 (30.9)		40 (72.7)	15 (27.3)		37 (67.3)	18 (32.7)	
LE	108	62 (57.4)	46 (42.6)		63 (58.3)	45 (41.7)		69 (63.9)	39 (36.1)	
cTNM stage				0.042*			0.035*			0.006**
II	82	44 (53.7)	38 (46.3)		45 (54.9)	37 (45.1)		45 (54.9)	37 (45.1)	
III	81	56 (69.1)	25 (30.9)		58 (71.6)	23 (28.4)		61 (75.3)	20 (24.7)	
pTNM stage				0.015*			0.020*			0.008**
II	97	52 (53.6)	45 (46.4)		54 (55.7)	43 (44.3)		55 (56.7)	42 (43.3)	
III + IV	66	48 (72.7)	18 (27.3)		49 (74.2)	17 (25.8)		51 (77.3)	15 (22.7)	
Differentiation				0.002**			0.002**			0.003**
Well (G1)	16	5 (31.3)	11 (69.7)		5 (31.3)	11 (69.7)		7 (43.8)	9 (56.2)	
Moderate (G2)	110	65 (59.1)	45 (40.9)		68 (61.8)	42 (38.2)		67 (60.9)	43 (39.1)	
Poor (G3)	37	30 (81.1)	7 (18.9)		30 (81.1)	7 (18.9)		32 (86.5)	5 (13.5)	
LVI				0.867			0.866			0.730
No	56	35 (62.5)	21 (37.5)		36 (64.3)	20 (35.7)		35 (62.5)	21 (37.5)	
Yes	107	65 (60.7)	42 (39.3)		67 (62.6)	40 (37.4)		71 (66.4)	36 (33.6)	

Nerve invasion				0.504			0.624			0.046
No	93	55 (59.1)	38 (40.9)		57 (61.3)	36 (38.7)		54 (58.1)	39 (41.9)	
Yes	70	45 (64.3)	25 (35.7)		46 (65.7)	24 (34.3)		52 (74.3)	18 (25.7)	
Tumor budding				0.182			0.065			0.137
Low	81	44 (54.3)	37 (45.7)		44 (54.3)	37 (45.7)		47 (58.0)	34 (42.0)	
Intermediate	65	44 (67.7)	21 (32.3)		47 (72.3)	18 (27.7)		48 (73.8)	17 (26.2)	
High	17	12 (70.6)	5 (29.4)		12 (70.6)	5 (29.4)		11 (64.7)	6 (35.3)	
pT stage				0.101			0.083			0.333
2	38	19 (50.0)	19 (50.0)		19 (50.0)	19 (50.0)		22 (57.9)	16 (42.1)	
3	125	81 (64.8)	44 (35.2)		84 (67.2)	41 (32.8)		84 (81.3)	41 (43.7)	
pN stage				0.045*			0.039*			0.033*
0	62	32 (51.6)	30 (48.4)		33 (53.2)	29 (46.8)		34 (54.8)	28 (45.2)	
1–3	101	68 (67.3)	33 (32.7)		70 (69.3)	31 (30.7)		72 (71.3)	39 (28.7)	

Note: *Significant difference. *p < 0.05, **p < 0.01.

Abbreviations: F, female; M, male; HPF, high power field; ME, middle esophagus; LE, lower esophagus; LVI, lymphatic invasion or venous invasion; PCC, positive cell count; PPH, PRDM1 positive hotspot.
